# Effect of Bioactive Glass-Containing Light-Curing Varnish on Enamel Remineralization

**DOI:** 10.3390/ma14133745

**Published:** 2021-07-04

**Authors:** Hyun-Jung Kim, So-Yeon Mo, Duck-Su Kim

**Affiliations:** 1 Department of Conservative Dentistry, Kyung Hee University Dental Hospital, Seoul 02447, Korea; kimhyunjung@khu.ac.kr; 2 Department of Conservative Dentistry, Graduate School, Kyung Hee University, Seoul 02447, Korea; iammosso@hanmail.net; 3 Department of Conservative Dentistry, School of Dentistry, Kyung Hee University, Seoul 02447, Korea

**Keywords:** enamel remineralization, varnish, bioactive glass, light-curing varnish, BAG varnish, dental materials, biomaterials

## Abstract

This study aimed to evaluate the effect of novel experimental light-curing bioactive glass (BAG)-containing varnish on enamel remineralization. An experimental light-curing, BAG-containing varnish and two commercial varnishes (Nupro White Varnish; Dentsply International, York, PA, USA and Tooth Mousse; GC Corporation, Tokyo, Japan) were used. Microhardness tests (n = 3), field emission scanning electron microscopy (FE-SEM) coupled with energy dispersive X-ray spectroscopy (EDS) (n = 5), and X-ray diffraction (XRD) analysis (n = 5) were performed to compare the remineralization effect of three varnishes with and without ultrasonication. The data of microhardness test were analyzed using one-way ANOVA and Tukey’s post hoc comparison (*P* < 0.05). Microhardness of demineralized enamel increased after the application of three varnishes (*P* < 0.05). The experimental BAG-containing varnish showed the highest microhardness among the three varnishes (*P <* 0.05). Ultrasonication decreased microhardness of Tooth Mousse and BAG-containing varnish groups (*P* < 0.05). FE-SEM and XRD revealed precipitates of hydroxyapatite (HAP) or fluorapatite (FAP) crystals of three varnishes. The novel experimental BAG-containing varnish may be a promising clinical strategy for the remineralization of early carious lesions or demineralized enamel surfaces.

## 1. Introduction

Enamel is the most mineralized tissue in the body, consisting of 95% (wt/wt) minerals, 4% (wt/wt) water, and 1% (wt/wt) organic material [1]. The inorganic content of enamel is composed of biological hydroxyapatite (HAP) crystals, Ca_10_(PO_4_)_6_(OH)_2_, and its rods are arranged into a highly organized prism [2,3]. Enamel has a higher inorganic content (~90%) than dentin, bone (~70%), and cementum (45%). With high inorganic content, the enamel is tougher and more resistant to force than any other hard tissue in the body. In a healthy oral environment, there is an equilibrium between demineralization and remineralization of tooth structure. A pronounced demineralization process could cause an initial pathologic change, ultimately leading to destruction of the tooth structure. This can be caused by local cariogenic bacteria in plaque (caries), non-bacterially derived erosive challenges (such as acidic beverages), or mechanical force [2]. Hard tissues in the body have different regeneration capabilities, except enamel, which shows no further biological activity [4].

However, since enamel regeneration is particularly challenging, remineralization, as a natural repair process, can only result in net mineral gain in demineralized lesions [5]. The remineralization process is usually regulated by saliva, remineralization solutions, or agents. Topical application of fluoride has long been practiced to arrest and remineralize initial or advanced demineralized enamel lesions [6]. It aids remineralization by forming fluorapatite (FAP) crystals on the enamel surface [7]. It has been incorporated in varnishes, gels, toothpastes, mouthwash, and other dental restorative materials. Casein-phosphopeptides (CPPs) with amorphous calcium phosphate (ACP) are the main components of commercially available anti-cariogenic agents [8]. CPP-ACP is based on milk-derived proteins and promotes remineralization of carious lesions by maintaining a supersaturated state of the enamel mineral [9]. In addition to these remineralization agents, various nanoparticles, such as silver, zinc oxide, titanium oxide, quaternary ammonium salt polyethylenimine nanoparticles, and nano-fluorapatite, have been studied for the prevention of early caries or enamel demineralization [10,11,12].

Bioactive glass (BAG) is a remineralization agent developed by Larry Hench in 1969. BAG has appealing characteristics as a scaffold material for bone tissue engineering [13]. It binds both soft and hard tissues and stimulates tissue mineralization owing to its superior bioactivity [14]. It has been adopted for medical use, such as for bone regeneration, and recent research has focused on its use in dental applications [15,16]. Several previous studies have indicated that BAG can remineralize enamel [17] and dentin [16] and has antimicrobial effect on oral bacteria [18].

Many studies have been conducted on BAG-containing experimental products in dentistry. BAG-containing dentin adhesive increases the nano-mechanical properties of demineralized dentin [19]. BAG releases calcium and fluoride ions when added to resin as a filler [20] and exhibits antibacterial activity [21] while maintaining the mechanical properties of the composite resin [22]. Placement of BAG-containing composites in close proximity to demineralized dentin can aid in remineralization [23]. In addition, BAG application can enhance the adhesion of resin-modified glass ionomers [24] and precipitate FAP crystals at the interface between demineralized dentin and BAG-incorporated GIC [25]. These studies indicate that presence of BAG in dental materials induces remineralization of the enamel and dentin without hampering its bioactivity [19,22,23].

Considering these bioactivities of BAG, it is assumed that it can be incorporated into the enamel varnish. Because most commercial varnishes applied on the enamel surface are easily removed by water rinsing, eating, or tooth brushing, more substantivity is necessary. Thus, a light-curable BAG-containing varnish was experimentally manufactured for this purpose. Light-cured varnish is advantageous because retention on the tooth surface is an important factor in delivering effective factors for remineralization of hard tissue [26].

The purpose of this study was to evaluate the effect of a novel experimental light-curing BAG varnish on enamel remineralization and to compare its efficacy with that of commercially available fluoride varnish and CPP-ACP varnish. In this study, microhardness, field emission scanning electron microscopy (FE-SEM) coupled with energy dispersive X-ray spectroscopy (EDS), and X-ray diffraction (XRD) were used for analysis.

## 2. Materials and Methods

### 2.1. Enamel Specimen Preparation and Experimental Materials

The study protocol was approved by the Institutional Review Board of the Kyung Hee University Dental Hospital (KHD IRB 1902-1). One hundred and four caries-free extracted human third molars were obtained for the preparation of enamel specimens. Enamel specimens (3.0 × 3.0 × 2.0 mm^3^) were fabricated using a high-speed saw (IsoMet 5000 Precision Saw; Buehler, Lake Bluff, IL, USA). The top surface of the specimen was ground using a fine diamond bur to obtain a flat enamel surface, which was then serially polished with 320, 400, 600, 800, 1200, and 1500-grit silicon carbide (SiC) paper. Phosphoric acid etchant, at 37% concentration (ETCH-37™; BISCO, Schaumburg, IL, USA), was applied to the enamel surfaces for 20 min to form a demineralized lesion, excluding the specimens assigned to the positive control group (undemineralized enamel).

Three varnishes were used in this study: (1) Nupro White Varnish (Dentsply International, York, PA, USA), a fluoride-based enamel remineralization agent; (2) Tooth Mousse (GC Corporation Tokyo, Japan), a CPP-ACP-based agent; and (3) novel BAG-containing experimental varnish. For the preparation of experimental BAG-containing varnish, BAG 63S (Bonding Chemical, Katy, TX, USA) was selected as the active component. It was developed using the sol-gel method with particle size less than 20 μm. The composition of these materials is listed in Table 1.

### 2.2. Experimental Groups

**Figure 1:**
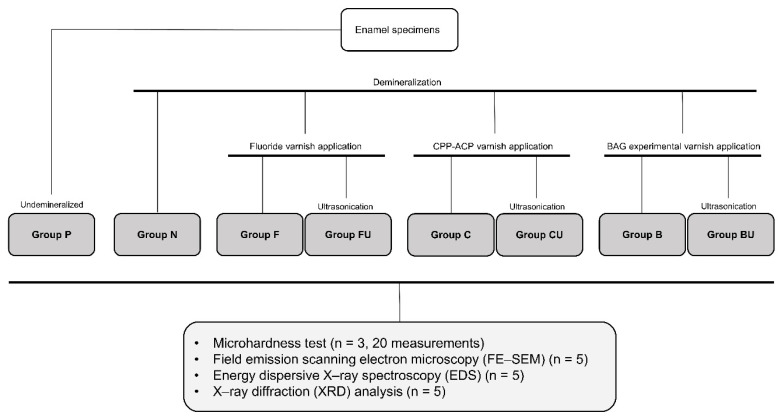
Classification of experimental groups and analysis methods.

Thirteen enamel specimens were randomly assigned to each of eight experimental groups according to the treatment modality (n = 13; Table 2 and Figure 1), including the positive control group (undemineralized; Group P) and negative control group (demineralized only; Group N). There were three experimental groups according to the type of varnish used, which were further divided into two subgroups based on whether the specimens underwent ultrasonication. Three of the thirteen specimens underwent the microhardness test, five underwent FE-SEM/EDS analysis, and the other five were used for XRD analysis. Two examiners (S.Y. Mo and D.S. Kim) who specialized in operative dentistry conducted the experiments.

Nupro White Varnish and Tooth Mousse were applied according to the manufacturer’s instructions (Table 1). The BAG-containing varnish was applied indirectly to the teeth because its removal could affect the remineralized enamel surface [23,25]. For indirect application of experimental varnish, blocks of composite resin (Any-Com; MEDICLUS, Cheongju, Korea) with dimensions of 6.0 × 6.0 × 4.0 mm^3^ were prepared as the BAG delivery medium. Then, BAG-containing varnish was applied onto the resin blocks with a microbrush and light-cured for 20 s. Subsequently, the resin blocks were approximated as close to the enamel specimens as possible with an orthodontic elastic band.

Nupro White Varnish and Tooth Mousse were rinsed with deionized water thoroughly for 30 s after application, and all specimens were stored in artificial saliva for 2 weeks. After storage, half the specimens in each experimental group, forming the groups FU, CU, and BU, underwent ultrasonification (Soniclean 160HT; Soniclean Pty Ltd., Thebarton, Australia) [27].

### 2.3. Microhardness Test

Three specimens from each experimental group were rinsed thoroughly with deionized water and were air-dried at room temperature. The microhardness of the enamel surface was measured using a VHN with a microhardness tester (HMV-2, Shimadzu Co., Kyoto, Japan) at 300 g for 5 s. The VHN was obtained using the following equation: VHN = 1854.4 P/d^2^, where P is the applied load in grams and d is the average length of the indentation measured in millimeters [6]. The VHN was measured at 20 different points of each specimen, and each measured point was at a constant distance from each other.

### 2.4. FE-SEM/EDS Analysis

Five specimens were used for the FE-SEM analysis in each experimental group. All specimens were stored in 20, 50, 75, 95, and 100% ethanol for 20, 20, 20, 30, and 60 min, respectively, for complete dehydration, and then air-dried at room temperature. The specimens were gold-sputtered and analyzed using FE-SEM (S-4700; Hitachi, Tokyo, Japan) at 10 kV. The same specimens were prepared for the detection of calcium, phosphorus, fluorine, and silicon using EDS analysis.

### 2.5. XRD Analysis

Five specimens were examined using an X-ray diffractometer (D8 advance; Bruker AXS, Karlsruhe, Germany) with Cu Kα radiation at 40 kV and 100 mA using a Ni filter. The diffraction intensities were measured by scanning in the range of 2*θ* (i.e., 20–60° in 0.01° steps for 0.1 s per step).

### 2.6. Statistical Analysis

The results of the microhardness tests of all experimental groups were analyzed by one-way ANOVA to reveal significant differences between the groups. Tukey’s test was used for post hoc comparisons. The level of significance was set at *P* = 0.05. All statistical analyses were performed using SPSS 23.0.0.0 (IBM Corp., Armonk, NY, USA).

## 3. Results

### 3.1. Microhardness Test

**Figure 2:**
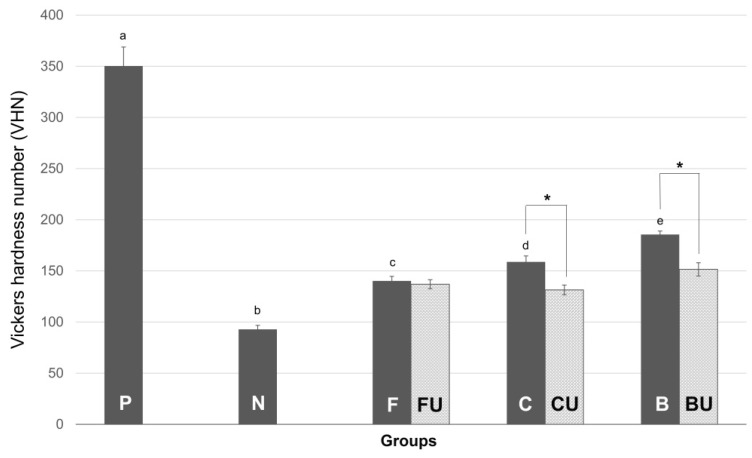
Results of the microhardness test. (n = 20). The unmineralized positive control (Group P) exhibited the highest Vickers harness number (VHN) and Group N the lowest. The VHN of all three varnish groups was greater than that of Group N. Among the three varnish groups, Group B showed the highest VHN, followed by groups C and F. The asterisk (*) indicates statistically significant differences between groups (*P* < 0.05).

The microhardness values of all experimental groups are shown in Figure 2.

Group P had the highest Vickers hardness number (VHN) among all experimental groups (*P* < 0.05), and Group N the lowest (*P* < 0.05). Groups F, C, and B had a higher VHN than Group N (*P* < 0.05). Group B had the highest VHN, followed by Groups C and F, among the three varnish groups. The differences among Groups F, C, and B were significant (*P* < 0.05).

The VHNs of Groups FU, CU, and BU were slightly lower than those of Groups F, C, and B, respectively, but higher than the VHN of Group N (*P* < 0.05). The difference in the VHN of Groups F and FU was not significant (*P* > 0.05), but the differences between Groups C and CU and between Groups B and BU were significant (*P* < 0.05). The VHN of Groups CU and BU was significantly different than that of Group FU (*P* < 0.05).

### 3.2. FE-SEM/EDS Analysis

**Figure 3:**
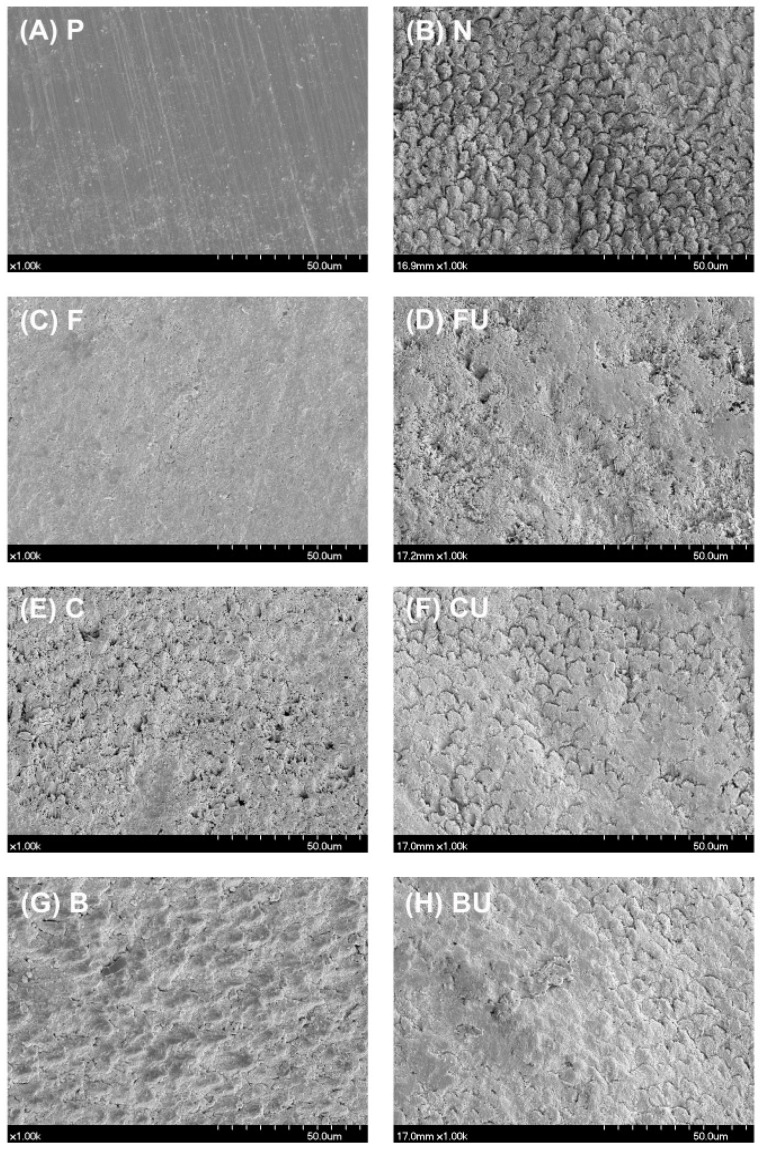
Representative FE-SEM images of all the experimental groups. (**A**) Group P, (**B**) Group N, (**C**) Group F, (**D**) Group FU, (**E**) Group C, (**F**) Group CU, (**G**) Group B, and (**H**) Group BU. Exposed enamel rods in Group N were covered with precipitates after the application of three varnishes with or without ultrasonication.

Representative FE-SEM images are shown in Figure 3.

**Figure 4:**
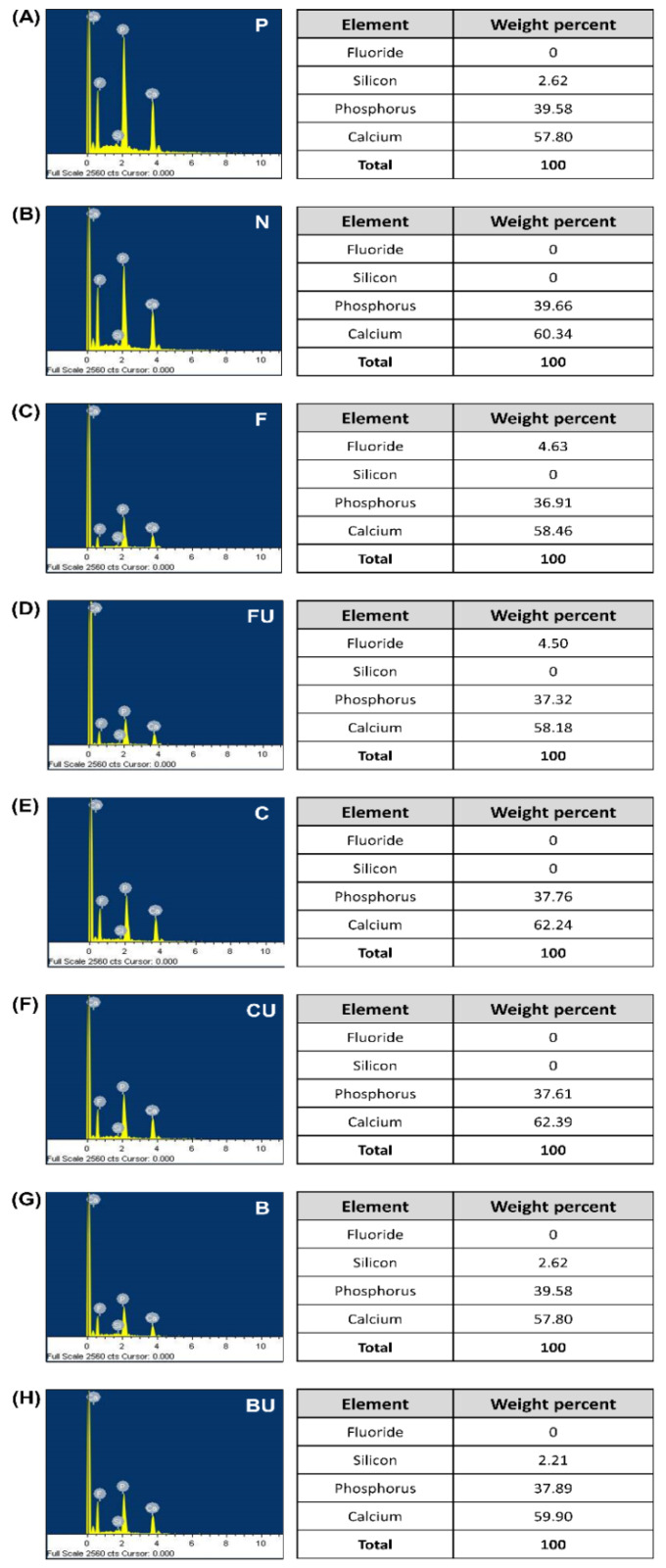
Results of EDS analysis. (**A**) Group P, (**B**) Group N, (**C**) Group F, (**D**) Group FU, (**E**) Group C, (**F**) Group CU, (**G**) Group B, and (**H**) Group BU. Calcium and phosphorus ions were detected in Group F and FU, and silica ion was in Group B and BU.

An enamel surface covered with a smear layer was observed in Group P (Figure 3A). After the enamel surface was demineralized, eroded enamel rods were observed in group N (Figure 3B). When each varnish was applied (Groups F, C, and B), demineralized enamel surfaces were covered with amorphous precipitates (Figure 3C,E,G). The morphology was similar to that seen in Group P. On performing ultrasonication (Groups FU, CU, and BU), a slight loss of these precipitates was observed, while most precipitates remained on the enamel surface (Figure 3D,F,H). The results of the EDS analysis for all experimental groups are shown in Figure 4.

Chemical characterization revealed that the phosphorus and calcium content was similar in all experimental groups. The Ca/P ratio was 1.4–1.6 in all experimental groups, similar to that of HAP. Calcium and phosphorus ions were the main components in Groups P and N (Figure 4A,B) [28]. Groups F and FU (Figure 4C,D) showed the presence of fluoride. Groups C and CU (Figure 4E,F) had a similar composition as that of the control groups, with no presence of fluoride or silicon. Groups B and BU (Figure 4G,H) contained silicon (2.62 and 2.21%, respectively) in addition to calcium and phosphorus.

### 3.3. XRD Analysis

**Figure 5:**
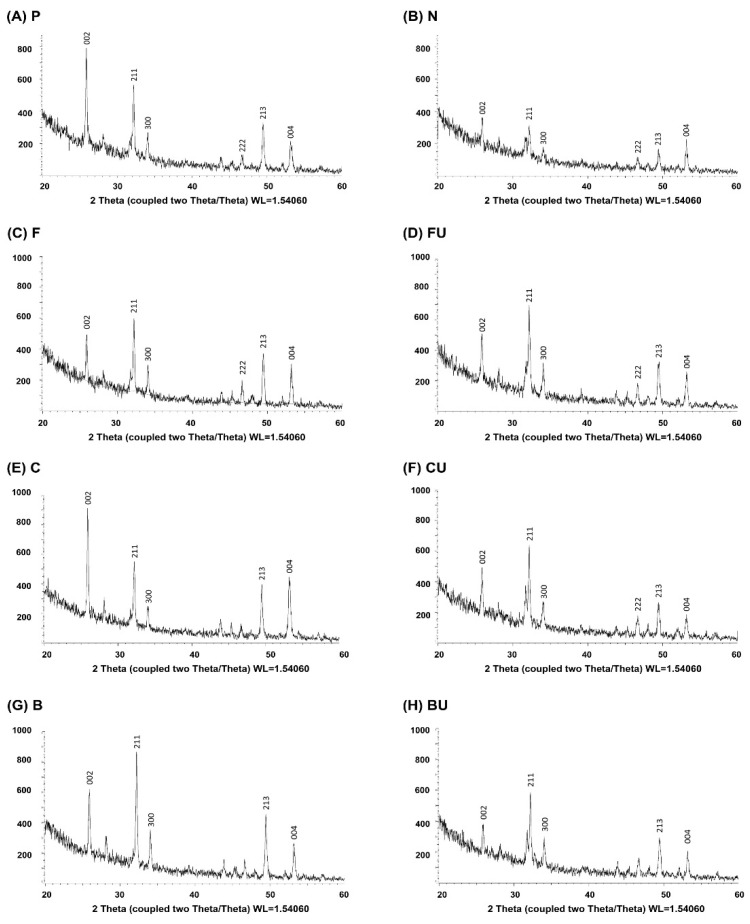
Results of XRD analysis. (**A**) Group P, (**B**) Group N, (**C**) Group F, (**D**) Group FU, (**E**) Group C, (**F**) Group CU, (**G**) Group B, (**H**) and Group BU. The decrease of HAP peaks [26° of (002) reflection and at 31.8° of (211) reflection] in Group N was obvious. It increased after the application of three varnishes with or without ultrasonication.

The results of the XRD analysis of all experimental groups are shown in Figure 5.

Group P showed intense peaks at approximately 26° of (002) reflection and at 31.8° of (211) reflection (Figure 5A). These peaks are representative of HAP crystals [29,30,31]. The intensity of these peaks was reduced in Group N (Figure 5B). The peak intensity increased after the application of varnishes (Figure 5C,E,G) and slightly decreased after ultrasonication, except for Group FU (Figure 5D,F,H).

## 4. Discussion

Remineralization of early carious lesions is naturally achieved by salivary ions or pH [32] and can be accelerated by adjunctive remineralizing agents [33]. Among them, fluoride-based agents are the most widely used and have the highest level of supporting evidence [34,35]. Although fluoride has a high affinity for HAP crystals, it has the disadvantage of forming FAP crystals only on the superficial layer because of its low penetration ability [36]. In addition, careless handling of fluoride can cause side effects, such as fluorosis [37]. At the population level, the effect of fluoride on prevention of dental caries has plateaued [38]. A significant remineralizing effect has been observed with CPP-ACP [8,39,40]; however, reviews have raised concerns about the excessive use of short-term in situ models, which have limited clinical reliability [33,41]. Additionally, most studies have been conducted on orthodontics-related incipient lesions, thus limiting the generalizability of the results to other carious lesions [33]. Consequently, the development of new agents has been attempted to overcome these limitations.

BAG can react with saliva, inducing the release of Ca^2+^, PO_4_^3−^, and Si^4+^ at the glass surface, and the subsequent precipitation of a polycondensated silica-rich layer serves as a template for the formation of calcium phosphate [42]. As the reaction and the deposition of Ca-P complexes continue, this layer crystallizes into hydroxycarbonate apatite, which is chemically and structurally similar to biological apatite. BAG was originally developed as a bone-regenerative material and has recently found use in oral care products, such as in toothpastes and mouth-rinses [43].

However, the application of BAG as a remineralization agent has been limited, owing to its relatively long reaction time. According to Hench, the release of calcium and phosphate ions from BAG requires at least 2 h [31]. Moreover, newly formed calcium and phosphate compounds from BAG were detectable after 24 h [44]. Originally, BAG was prepared by the melt-quenching method, and its particle size was considerably large. Because of the large particle size, it was not easy to incorporate BAG into dental materials. Thus, the sol-gel method was developed to prepare BAG to reduce the particle size and increase bioactivity [45]. It is known that BAG made by the sol-gel method has higher porosity levels and a larger surface area than BAG made by the melt-quenching method [46]. These porous structures can increase the bioactivity of BAG [47]. Based on these results, the BAG used in this study was prepared using the sol-gel method, to enhance bioactivity.

Numerous studies have been conducted on the enamel remineralization effect of BAG. The 45S5 BAG paste reportedly improved the microhardness of the subsurface eroded enamel surface [6]. Application of BAG paste to incipient enamel erosive lesions was seen to restore lesions with an abrasion durable layer of HAP [48]. BAG-containing toothpaste had a greater effect on the remineralization of carious-like lesions in permanent teeth compared to that of fluoride-containing toothpaste [49]. In addition, BAG showed remineralization of caries in primary teeth similar to that by 500 ppm fluoride toothpaste and CPP-ACP [50]. As reported in previous studies, BAG is mostly used as a paste. Unfortunately, this form does not have sufficient substantivity on the enamel surface. Therefore, to increase the application time, a light-curing BAG-containing varnish was designed and used in this study. In a previous study of a light-curable fluoride varnish, it was suggested that the varnish can provide protection for up to 4 months or even longer [26]. Longer retention of varnish might enable a decrease in the frequency of application and maximize the remineralizing effect with a single application in clinics. To the best of our knowledge, a light-curing BAG varnish has not yet been developed and reported in dentistry, although experimental BAG varnish without a curing procedure has been introduced [51]. In a previous study, the experimental BAG varnish showed that BAG formed apatite at 0, 3, 6, 24, 72, and 168 h of immersion. The longer it was immersed, the more ion release of the BAG-incorporated varnish was reported with more dissolution of Ca^2+^, Sr^2+^, PO_4_^3−^, and F^−^ [51].

In this study, we confirmed that the experimental BAG varnish regained the highest microhardness after demineralization compared to the other experimental groups (Groups F and C) (Figure 1). The microhardness of remineralized groups (Groups F, C, and B) was significantly lower than that of the positive control (Group P) but higher than that of the complete mineralized negative control (Group N). Ultrasonication decreased the microhardness of the remineralized groups, except that of Group F (*P* < 0.05), but not the microhardness of the negative control (Group N). The VHN was measured at 20 different points on each specimen, and each measured point was at a constant distance from each other. This was to control the variation of tooth substrate, so that 20 measurements were conducted in one specimen; conclusively, 60 VHN results were acquired in each group.

FE-SEM analysis revealed amorphous precipitate deposition on the enamel surface, except in the control groups (Groups P and N) (Figure 3). XRD analysis showed representative peaks at approximately 26° of (0 0 2) reflection and 31.8° of (2 1 1) reflection, which indicates HAP crystals in all the experimental groups except Group N (Figure 5) [29,30,31]. HAP and FAP have similar diffraction patterns and are known to be difficult to identify through XRD data because their lattice parameters are very close and structurally similar [52]. However, in Groups F and FU, it was assumed that both peaks represented FAP. Formation of FAP is preferred over the production of HAP in a fluoride-containing environment because FAP is chemically more stable and shows less dissolution at low pH [12,53]. EDS spectra confirmed the presence of fluoride in Groups F and FU, but not in the other groups (Figure 4). Based on these experimental results, it was suggested that the remineralizing agent, including BAG-containing experimental varnish, can deposit HAP or FAP crystals on the demineralized enamel surface; in particular, the experimental varnish showed the highest recovery of microhardness (*P* < 0.05).

In this study, ultrasonication was performed to confirm the substantivity of the newly formed layer. According to the results, most of the precipitates remained on the enamel surfaces. This suggests that the newly formed layer could chemically interact with the underlying enamel or that precipitates tended to be retained in the demineralized lacunae. The durability of the crystalline layer formed by the application of BAG had been confirmed in previous studies; the layer showed resistance to brushing-abrasion and transformed to HAP crystals in the remineralizing solution after 14 days [48].

BAG-containing varnish was applied indirectly to the specimens for the easy removal of varnish in this study. Direct application is more similar to clinical situations. In case of direct application, removal of varnish might deform the enamel surface, which is presumed to be remineralized.

The BAG-containing varnish has clinical advantages, such as immediate adhesion to the enamel surface by light curing and sufficient reaction time. Even if it is expected to have long sustainability, it is inevitable that it might be partly removed by brushing and chewing. Nevertheless, it could persist for a longer time than conventional varnishes, and it is predicted that the remineralization process would be more stable. Moreover, silica glass particles might impart wear resistance to the experimental BAG-varnish before mechanical deformation. It was reported that a dental varnish with high filler content can resist up to 5000 strokes of brushing [26].

Factors affecting the remineralization ability of BAG-containing varnish include the amount of BAG and the substantivity of varnish. If more BAG content and longer application time are guaranteed, more remineralization might occur. However, an excessive amount of BAG may change the mechanical properties of the varnish. Therefore, further studies are necessary to determine the optimal amount of BAG and the optimal duration of application.

This study examined the effect of a novel light-curing BAG-containing varnish on enamel remineralization. The varnish exhibited superior recovery of microhardness in demineralized enamel and showed equal or better HAP crystal precipitation compared to commercial dental varnishes. It may be adopted for early enamel lesion treatment, can persist for a longer time than conventional varnishes, and can be applied more conveniently. The experimental light-curing BAG-varnish can provide protection for a relatively long application time and may also reduce the frequency of patient visits for preventive dental care.

Although this study proved the effectiveness of novel BAG-containing varnish on enamel remineralization in vitro, further studies are necessary to determine the optimal content of BAG and retention period of BAG-containing varnish on enamel. In addition, for clinical application of the BAG varnish, in vivo tests and clinical trials will be necessary.

## 5. Conclusions

We conclude that BAG-containing varnish can help regain the microhardness of demineralized enamel and increase HAP crystal precipitation on the demineralized surface. The BAG-containing varnish has clinical advantages, such as immediate adhesion to the enamel surface by light curing and sufficient reaction time. The newly formed HAP layer was relatively stable under ultrasonication conditions.

The application of light-curing experimental BAG varnish could be a useful strategy to promote remineralization of early carious lesions or demineralized enamel surfaces in minimally invasive dentistry with a prolonged application time and greater convenience of use.

## Figures and Tables

**Table 1:** List of varnishes used in this study.

Materials	Composition	Applications
Fluoride varnish (Nupro White; Dentsply international, York, PA, USA)	Urethane Dimethacrylate Resin 30–40 wt% 2-Propanol 20–30 wt% Sodium Fluoride 5 wt% Titanium Dioxide <1 wt%	Apply once (2 h)
CPP-ACP Varnish (Tooth Mousse; GC Corporation Tokyo, Japan)	CPP-ACP 10 wt% Glycerol 20 wt% D-Sorbitol 5 wt% Silicon dioxide 2 wt% Propylene glycol 2 wt% Titanium dioxide 2 wt% Ethyl-p-hydroxybenzoate <0.1 wt% Butyl-p-hydroxybenzoate <0.1 wt% Propyl-p-hydroxybenzoate <0.1 wt%	Apply daily for 7 days (30 min per day)
BAG-containing Varnish (Experimentally manufactured)	63S Bioglass 30 wt% UDMA (Diurethane dimethacrylate) 31.36 wt% HEMA (Ethylene glycol methacrylate) 9.24 wt% CQ (Camphorquinone) 0.35 wt% EDMAB (ethyl-4-dimethylaminobenzoate) 0.7 wt% BHT (2,6-di-tert-butyl-4-methylphenol) 0.175 wt% TP (2,2-(P-Tolylimino)-diethanol) 0.175 wt% EtOH 28 wt%	Indirect application; Light curing (20 s) after applying the varnish on composite resin block and fixing the applied surface and enamel surface in tight contact

**Table 2:** Protocol for each experimental group.

Groups	Description	Procedures
Group P	Positive control (Undemineralized enamel)	Untreated
Group N	Negative control (Demineralized enamel)	Demineralization (37% phosphoric acid, 20 min)
Group F	Nupro White Varnish	Demineralization (37% phosphoric acid, 20 min) → Nupro White Varnish application (once, 2 h) → Storage (artificial saliva, 2 weeks)
Group FU	Nupro White Varnish + Ultrasonication	Demineralization (37% phosphoric acid, 20 min) → Nupro White Varnish application (once, 2 h) → Storage (artificial saliva, 2 weeks) → Ultrasonication (3 min)
Group C	Tooth mousse	Demineralization (37% phosphoric acid, 20 min) → Tooth mousse application (for 7 days, 30 min per day) → Storage (artificial saliva, 2 weeks)
Group CU	Tooth mousse + Ultrasonication	Demineralization (37% phosphoric acid, 20 min) → Tooth mousse application (for 7 days, 30 min per day) → Storage (artificial saliva, 2 weeks) → Ultrasonication (3 min)
Group B	BAG-containing varnish	Demineralization (37% phosphoric acid, 20 min) → BAG varnish application (indirectly, light curing 20 s) → Storage (artificial saliva, 2 weeks)
Group BU	BAG-containing varnish + Ultrasonication	Demineralization (37% phosphoric acid, 20 min) → BAG varnish application (indirectly, light curing 20 s) → Storage (artificial saliva, 2 weeks) → Ultrasonication (3 min)

## Data Availability

The data presented in this study are available on request from the corresponding author.
